# Supplementary figures and images for: Risk Factors for Postural and Functional Balance Impairment in Patients with Chronic Obstructive Pulmonary Disease
Source: J Clin Med. 2020 Feb 24;9(2):609. doi: 10.3390/jcm9020609 (PMC7074538; doi:10.3390/jcm9020609)

## Berg Balance Scale

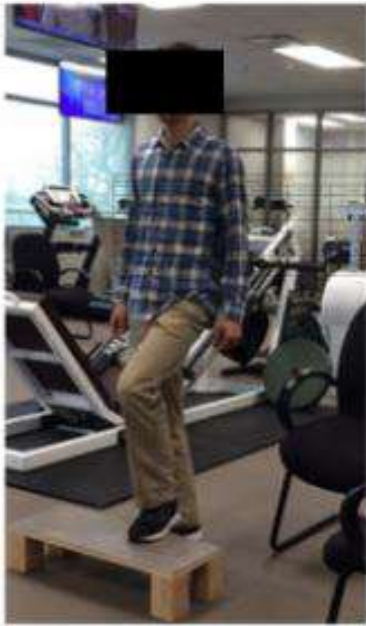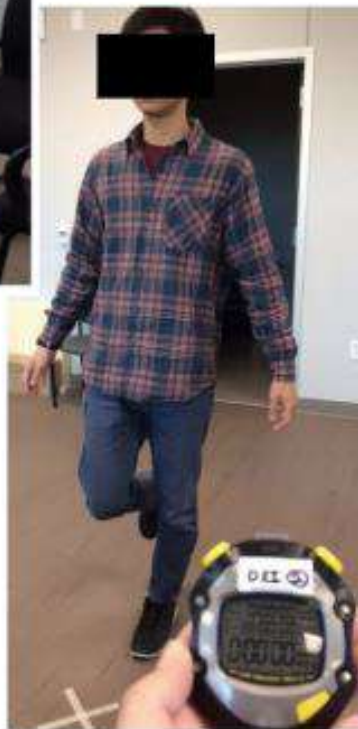

## CoP measurement during QS

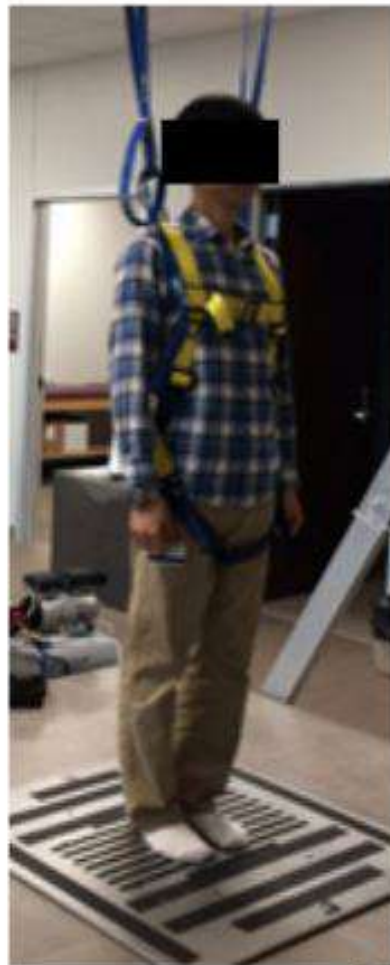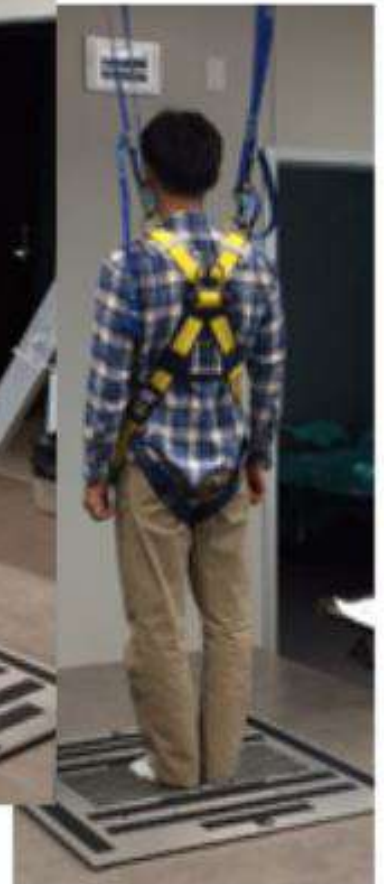

Supplement: Supplementary file 1 [file jcm-09-00609-s001.pdf]
